# Pain perception during social interactions is modulated by self-related and moral contextual cues

**DOI:** 10.1038/s41598-019-56840-x

**Published:** 2020-01-08

**Authors:** Valentina Nicolardi, Maria Serena Panasiti, Mariagrazia D’Ippolito, Gian Luigi Pecimo, Salvatore Maria Aglioti

**Affiliations:** 1 Department of Psychology, Sapienza University of Rome, Rome, Italy; 2 Fondazione Santa Lucia, IRCCS, Rome, Italy; 3 Istituto Italiano di Tecnologia, Genova, Italy

**Keywords:** Pain, Human behaviour

## Abstract

Despite the growing interest on the effect of the social context on pain, whether and how different facets of interpersonal interactions modulate pain are still unclear. We tested whether personal (i.e., convenient for the self), moral (i.e., equitability of the transaction) or social (i.e., positive vs. negative feedback from others) valence of an interpersonal interaction differentially affects pain and the perceived fairness. Thirty-two healthy participants played the role of Receivers in a Dictator Game, where a player, the Dictator, determined how to divide a payoff between her/himself and the other player, the Receiver. We manipulated the payoff (pain vs. money), the personal valence (favorable vs. unfavorable offer to participants), the moral valence of the offer (from very iniquitous to equitable), and social valence of the Dictator (social acceptance vs. rejection). Moral and personal valence differentially modulated pain. Lower pain was elicited by iniquity, but also by favorable offers. Moreover, unfavorable offers in the economic game were rated as more unfair, whereas only very iniquitous offers elicited such ratings in the pain game, suggesting that participants valued when Dictators endured extra pain for their benefit. Together, we show that the valence of a social interaction at different levels can independently modulate pain and fairness perception.

## Introduction

Mounting evidence suggests that not only cognitive and emotional^1–4^, but also social^5,6^ factors modulate the experience of pain. Although inducing negative emotional states causes an increase in pain perception^7–9^, the effects of social interactions on pain are less clear^10,11^. Experiencing a negative outcome during a social interaction can decrease pain sensitivity when the interaction is mildly negative (i.e., standoffish behavior by the partner)^12–14^. Moreover, studies about how a threatening context (intentional/unintentional harm inflicted by a confederate) can affect pain provided conflicting results even when performed by the same group^15,16^. Differences in designs, samples, dependent variables and experimental manipulations, makes it difficult to compare all the above studies. However, even considering only studies on healthy participants, which used similar tasks to manipulate the interpersonal context^14–18^, we observe crucial differences in the way in which context is conceived. The valence of the interaction, for example, was manipulated at different levels. Mancini and colleagues^14^ manipulated the moral valence (i.e., moral dispositions based on a set of internalized constraints regarding what is considered good or bad^19,20^), and found a pain reduction after unfair behavior of the partner. Story and colleagues^18^ also find a pain reduction but only when the partner favored the participant, as they manipulated the personal valence (i.e., whether a specific situation is favorable or unfavorable to the self in comparison to the other person^21,22^). Karos and colleagues^15,16^, instead, as in other studies^8,12,23^, manipulated the social valence (i.e., positive or negative attitude of the interacting partner toward the participants) and found a pain increase when it was related to a partner’s negative attitude. Previous studies showed that each social factor can plays a differential role in the way the interpersonal context modulates pain. Moreover, modulations may go in different directions depending on the factor manipulated. However, none of the above studies considered all these factors together within the same paradigm. Thus, having many different sources of variance may explain the conflicting results.

## Aims

The aim of this study was to try and reconcile some of the discrepancies in the existent literature regarding the influence of moral, personal and social variables on pain perception. To do this, we developed a novel economical paradigm based on different versions of the Dictator Game, a game in which a player, the Dictator, allocates an object (e.g., money) at will, while another player, the Receiver, can only accept the decision. Such bargaining games were initially used in the field of economics to test the profit maximization hypothesis, which proposes that self-interest is the basis of economic decisions^24^. However, neuroscientific studies have employed bargaining games to investigate the effect of perception and interoception on participants’ behavior during games^25,26^ and manipulate the context to test its effect on participants’ perception^14,18^. This study aimed to distinguish between different contextual variables on pain by devising a task that allowed us to manipulate the interaction object (i.e., money or pain), the social valence (i.e., a person who positively or negatively evaluates participants), the personal valence (i.e., favorable offer: more money and less pain to the participant compared to the other player, or unfavorable offer: less money and more pain to the participant compared to the other player), and the moral valence (i.e., objective equity) of an offer. Participants were always assigned to the role of Receiver, while the Dictator’s behavior was simulated by the pc and justified through a cover story (see Supplementary Information). In order to manipulate the personal and moral valence of each offer we variate the apportion of money/ pain between the two participants. Concerning the social valence, a mock social feedback indicating social acceptance or rejection by the Dictators, has been provided to the participants through an interpersonal manipulation (see Supplementary Information). In order to modulate the valence of the object of the offer as well, we included both versions of the game: the economic (money as payoff), and the pain (pain as payoff) version respectively.

## Results

### Pain perception

**Figure 1:**
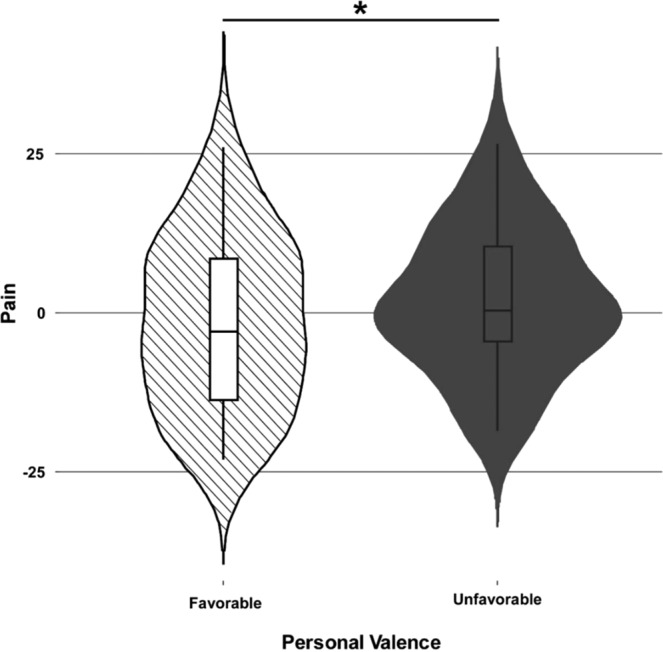
Main effect of personal valence on pain perception. The violin plots show the distribution of the dependent variable (i.e., pain), which is centered at the mean. Each plot shows the distribution density of the mean by subject for each condition. The central boxplot indicates the mean and standard deviation. The results show higher pain ratings when an offer was unfavorable compared to favorable (p = 0.031, χ^2^ = 4.6193). Favorable offers are hatched, and unfavorable offers are dark grey.

**Figure 2:**
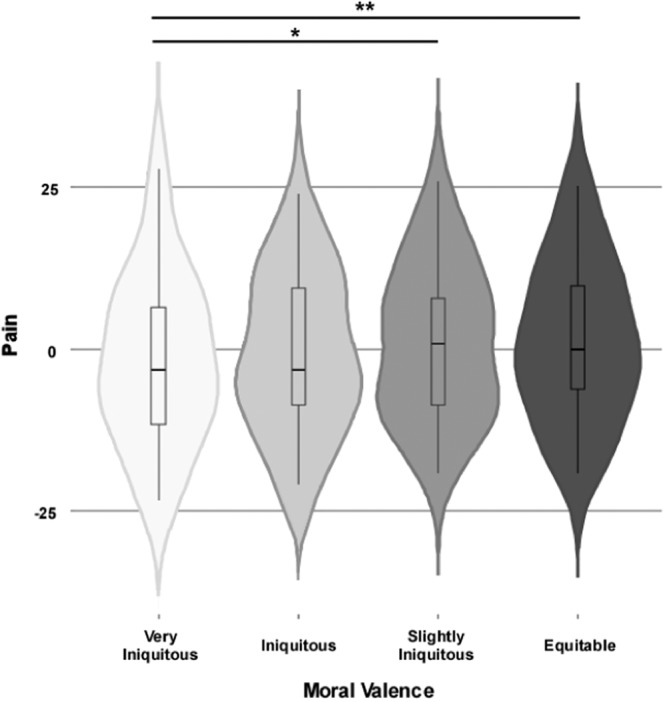
Main effect of moral valence on pain perception. Violin plots show the distribution of the dependent variable (i.e., pain), which is centered at the mean. Each plot shows the distribution density of the mean by subject for each condition. The central boxplot indicates the mean and standard deviation. The results show a significant pain increase between very iniquitous and slightly iniquitous or equitable offers (p < 0.0001, χ^2^ = 19.4775). White: very iniquitous; Light grey: iniquitous; Intermediate grey: slightly iniquitous; Dark gray: equitable.

Table 1 shows the results for subjective pain ratings. We report a lack of significance for the social rejection vs. acceptance manipulation (social valence: p = 0.33, χ^2^ = 3.386) and no significant interactions between the regressors. However, we found effects for personal and moral valence. Pain ratings varied significantly with the personal valence of an offer (p = 0.031, χ^2^ = 4.619) and were higher when an offer was unfavorable compared to favorable (Fig. 1). Moreover, the level of moral valence predicted significant changes in pain perception (p = 0.00021, χ^2^ = 19.477), with more equitable offers associated with greater reported pain (Fig. 2).

**Table 1:** Analysis of deviance (type III Wald chi-square tests) of subjective pain ratings.

	Chi-sq	Df	p
Intercept	3.3869	1	0.065
**Personal Valence**	**4.6193**	**1**	0.031*
**Moral Valence**	**19.4775**	**3**	<0.0001***
SocialValence:PersonalValence	0.2849	1	0.593
SocialValence:Moral Valence	6.3367	3	0.096
Personal Valence:Moral Valence	4.0690	3	0.254
SocialValence:Personal Valence:Moral Valence	4.6446	3	0.199

Significance codes: 0 ‘***’ 0.001 ‘**’ 0.01 ‘*’ 0.05 ‘.’ 0.1. Analysis of deviance of subjective pain ratings performed with a type III Wald chi-square test. Values in bold indicate significant results for Model 1: Pain ~ Social Valence * Personal Valence * Moral Valence + *(Social Valence* + *Personal Valence* + *Moral Valence* + *Social Valence:Personal Valence* + *Social Valence:Moral Valence* + *Personal Valence:Moral Valence|Subj). On the right columns the table shows the Chi-squared (Chi-sq), the degree of freedom (Df) and the level of significance* (p).

### Perceived fairness

**Figure 3:**
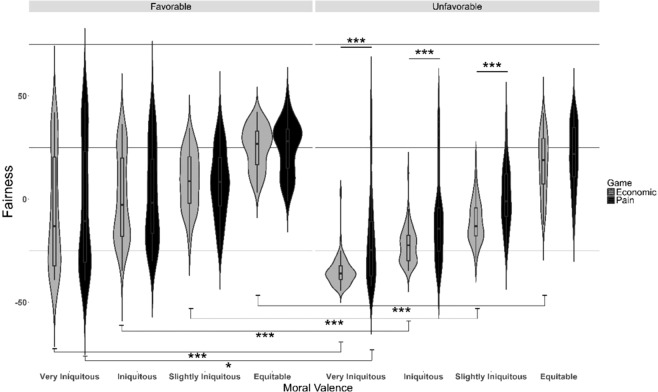
Significant personal valence (i.e., favorable (F): more money and less pain, or unfavorable (U): less money and more pain) * moral valence (very iniquitous, iniquitous, slightly iniquitous, or equitable) * game (money or pain offers) interaction for fairness ratings. Each plot shows the distribution density of the mean by subject for each condition. The central boxplot indicates the mean and standard deviation. Bold lines with asterisks (top panel) show how ratings differ between the two games for unfavorable and inequitable offers. Lines with asterisks (bottom panel) show that the favorable (F) and unfavorable (U) difference is significant at every level of moral valence in the economic game, whereas, in the pain game, it is significant only for very iniquitous offers. Grey and black violins represent the Economic Dictator Game and Pain Dictator Game, respectively.

Models 2 and 3 show the same significance effects, except for social valence, which was not significant in Model 2 (social valence, p = 0.11). To avoid redundancy, we will present the fairness results from Model 3. Table 2 shows the results for the perceived equity ratings. We found that personal and moral valence significantly predicted the perceived fairness. Interestingly, we also found a triple interaction between the tested factors. For the main effects of personal (p < 0.0001, χ^2^ = 40.793) and moral (p < 0.0001, χ^2^ = 35.137) valence, the perceived fairness increased with the increasing equity of an offer and decreased with decreasing favorability of an offer (i.e., lower perceived fairness for inconvenient vs. convenient offers) at each level of moral valence (personal valence * moral valence interaction: p < 0.0001, χ^2^ = 33.521; Fig. 3, grey violins). The triple interaction of personal valence * moral valence * game (Fig. 3, p = 0.015, χ^2^ = 10.346) demonstrated that, there was a significant difference in perceived fairness between the two games (Table 3) for unfavorable offers (Fig. 3, right panel) at every level of moral valence, excluding the Equitable offers. In the economic game, the unfavorable offer was rated as more iniquitous than that in the pain game (Table 3, Bold Grey). Moreover, in the pain, but not the economic, game the difference between favorable and unfavorable offers was significant only for very iniquitous offers. Finally, in the economic game, the difference between favorable and unfavorable offers was always significant.

**Table 2:** Analysis of deviance (type III Wald chi-square tests) of fairness ratings.

	Chi-sq	Df	p
Intercept	18.987	1	0.168
**Personal Valence**	**40.793**	**1**	<0.0001***
**Moral Valence**	**35.137**	**3**	<0.0001***
Game	0.3474	1	0.555
**Personal Valence:Moral Valence**	**33.521**	**3**	<0.0001***
**Personal Valence:Game**	**4.368**	**1**	0.004 **
Moral Valence:Game	2.122	3	0.547
**Personal Valence:Moral Valence:Game**	**10.346**	**3**	0.015*

Analysis of deviance of fairness ratings performed with a type III Wald chi-square test. The bold lines indicate significant results for Model 3: Fairness (Both Dictator Games) ~ Personal Valence * Moral Valence * Game + *(Personal Valence* + *Moral Valence* + *Game* + *Personal Valence:Moral Valence* + *Personal Valence:Game* + *Moral Valence:Game|Subj). On the right columns the table shows the Chi-squared (Chi-sq), the degree of freedom (Df) and the level of significance* (p).

**Table 3:** Post-hoc Comparisons for PERSONAL VALENCE * MORAL VALENCE * GAME.

contrast	estimate	SE	df	z.ratio	p.value
**Moral valence: very iniquitous**					
F,1 - U,1	29.466	4.197	Inf	7.020	<0.0001
**F,1 - F,2**	**−2.693**	**2.975**	**Inf**	**−0.905**	**0.802**
F,1 - U,2	13.759	4.69	Inf	2.931	0.0178
U,1 - F,2	−32.160	4.796	Inf	−6.705	<0.0001
**U,1 - U,2**	**−15.707**	**2.651**	**Inf**	**−5.924**	**<0.0001**
F,2 - U,2	16.453	5.892	Inf	2.792	0.0269
**Moral valence: iniquitous**					
F,1 - U,1	22.953	3.369	Inf	6.813	<0.0001
**F,1 - F,2**	**−1.449**	**2.975**	**Inf**	**−0.487**	**0.9620**
F,1 - U,2	11.457	3.677	Inf	3.115	0.0099
U,1 - F,2	−24.402	3.984	Inf	−6.124	<0.0001
**U,1 - U,2**	**−11.496**	**2.651**	**Inf**	**−4.336**	**0.0001**
F,2 - U,2	12.906	5.035	Inf	2.563	0.0508
**Moral valence: slightly iniquitous**					
F,1 - U,1	19.947	2.928	Inf	6.811	<0.0001
**F,1 - F,2**	**0.857**	**2.975**	**Inf**	**0.288**	**0.9917**
F,1 - U,2	7.689	2.674	Inf	2.875	0.0211
U,1 - F,2	−19.089	3.005	Inf	−6.352	<0.0001
**U,1 - U,2**	**−12.257**	**2.651**	**Inf**	**−4.623**	**<0.0001**
F,2 - U,2	6.832	3.861	Inf	1.769	0.2881
**Moral valence: equitable**					
F,1 - U,1	8.564	2.510	Inf	3.412	0.0036
**F,1 - F,2**	**0.558**	**2.975**	**Inf**	**0.188**	**0.9977**
F,1 - U,2	4.255	1.747	Inf	2.435	0.0706
U,1 - F,2	−8.005	2.065	Inf	−3.877	0.0006
**U,1 - U,2**	**−4.308**	**0.651**	**Inf**	**−1.625**	**0.3644**
F,2 - U,2	3.697	0.884	Inf	1.282	0.5744

Post-hoc comparisons for the triple interaction between personal valence * moral valence * game. Comparisons are listed according to moral valence level (very iniquitous, iniquitous, slightly iniquitous, and equitable). Bold grey values indicate comparisons of favorable (F) or unfavorable (U) offers within the same game, whereas bold black values denote comparisons of the economic (1) or pain (2) games within the same valence category. The results are averaged over the level of social valence. P-values were adjusted according to the Tukey method to compare a group of four estimates.

### Manipulation check

**Figure 4:**
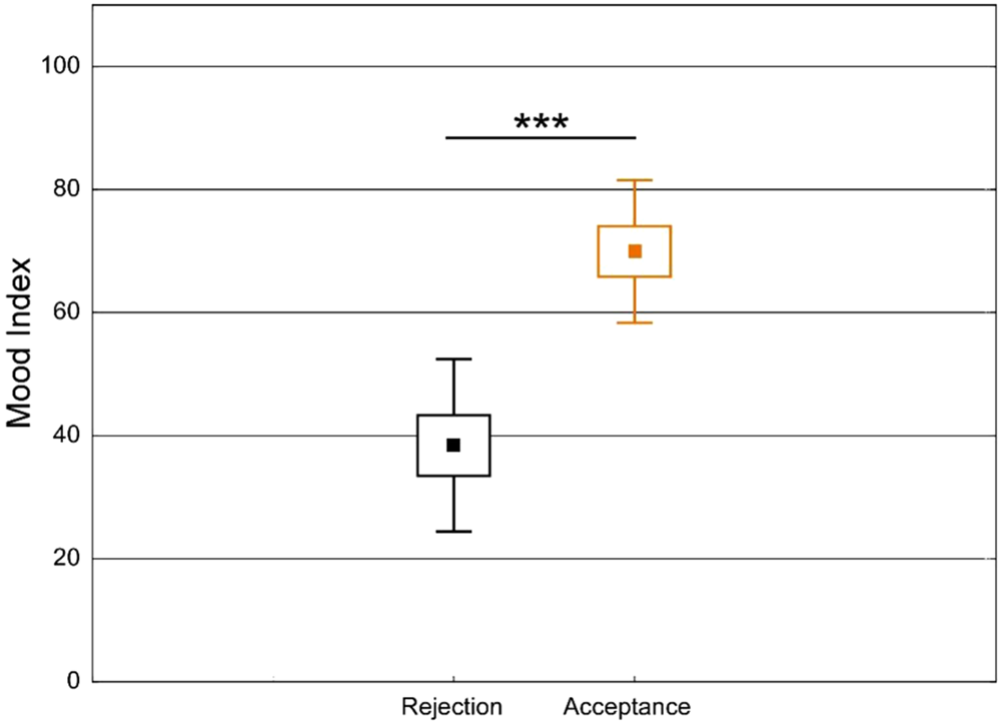
Differences in reported mood index following partner feedback. The Mood Index reported after a negative (Rejection) or a positive (Acceptance) feedback are represented by the black and orange boxes respectively. Lines indicate standard deviations while central boxes represent the mean value.

**Figure 5:**
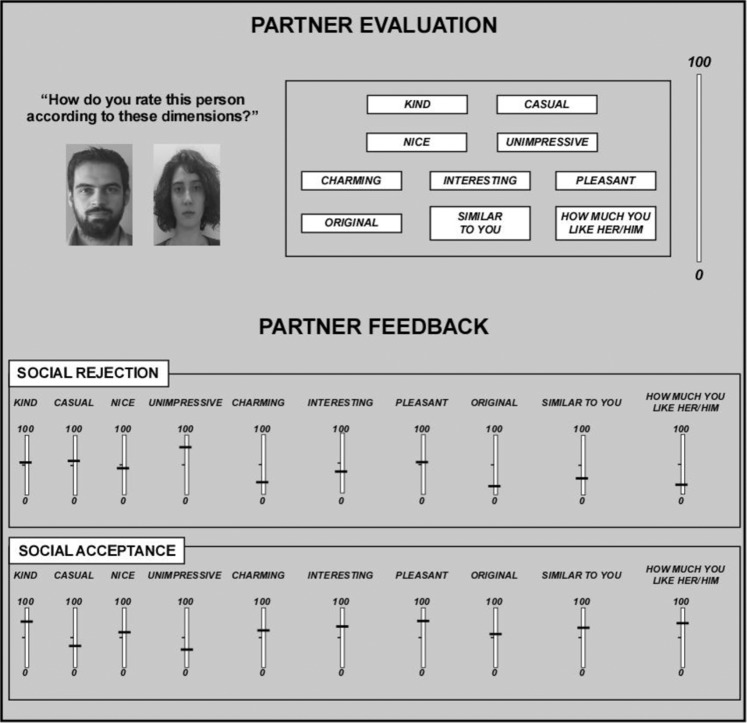
Interpersonal manipulation. The two phases illustrated in the figure (i.e., partner evaluation (top) and partner feedback (bottom)) sought to manipulate social valence by inducing a sense of social rejection or acceptance. In the partner evaluation phase, participants were asked to evaluate their partners on the 10 dimensions listed in the figure. In the partner feedback phase, participants were provided with false partner feedback as shown in the figure. False feedback was either mildly negative (i.e., social rejection) or positive (i.e., social acceptance).

To control for possible confounds due to the inclusion of non-believers, we tested the mixed-models by only including participants who believed what they were told about the experiment (n = 28). With the inclusion of only believers, all the effects previously reported remained unchanged. Moreover, we found a significant difference in the mood reported following partner feedback (Fig. 4, p < 0.0001), with a lower mood reported after rejection compared to acceptance (see Supplementary Information and Fig. 5).

## Discussion

To explore how the specific valence of interpersonal interactions influences pain and fairness ratings, we used two versions of the Dictator Game, in which we manipulated the object (i.e., money or pain) of an offer, the social valence of the interaction (i.e., social rejection or acceptance feedback from the Dictator), the personal valence (i.e., favorable: more money or less pain, or unfavorable: less money or more pain); and the moral valence (i.e., very iniquitous, iniquitous, slightly iniquitous, or equitable) of the offer. Our findings showed that the moral and personal valence of an offer affect pain perception and perceived fairness. Specifically, pain decreased for either very iniquitous or favorable offers, whereas fairness ratings were higher for favorable offers, particularly when the object of the transaction was money rather than pain. The interpersonal manipulation procedure did not influence ratings of pain or offer fairness.

Moral and personal valence significantly influenced pain ratings. Regarding moral valence, an allocation of an equitable proportion of pain by the Dictator triggered higher pain perception, whereas more iniquitous offers were associated with pain reduction. It is possible that iniquitous offers garnered more attention and, thus, reduced the resources available to process painful stimuli. Aversion to unequal resource distribution between individuals^20^ is a wide-spread phenomenon that is also present in non-human primates^27^ and appears very early in human development^28^. As such, inequity is likely to attract attention and engage cognitive resources^29,30^. In a previous study we found a decrease in pain perception when a loss was inequitable (i.e., caused by unfair behavior by another player) and suggested that attentional engagement exerted by the inequitable outcome drove this effect, leading participants to be distracted from pain^14^. For the present study, we posit that the same mechanism could be at play. Importantly, we have significantly expanded previous knowledge by showing that pain reduction is due to inequity and not to the personal favorability of an offer. A recent Dictator Game study^18^ failed to find an effect of moral valence on pain perception; however, it found a significant effect of knowing that the other player was enduring extra pain compared to the participant. This interaction was apparent with iniquitous offers in which one player received fewer painful stimuli than the other player (e.g., the participant receives six shocks, whereas the other player receives 18 shocks). In contrast to our study, the authors did not consider the extent of iniquity as a factor. However, they were better able to distinguish between convenience (advantageous for the participant, what we called personal valence) and intentionality (if the Dictator’s choice was deliberate or not, a variable that we did not manipulated), including advantageous or disadvantageous, and intentional or chance allocations of pain. Instead, we focused on the distinction between the convenience (i.e., personal valence) and equitability (i.e., moral valence) of an offer. Moreover, the high variability between Receivers and Dictators may account for why Story *et al*.^18^ did not find an effect using a whole-sample analysis. Our result is also in agreement with non-human animal studies that report hypoalgesic responses to social stressors, including social isolation^31^, defeat experiences^32^ and social conflict^33^. However, another possible explanation might be suggested by the evolutionary theory, according to which, emotions and pain are expressed when it is advantageous to do it (for examples when one calls for help from others^34^). On the other hand, expressing emotions and pain in an adverse and threatening context might signal vulnerability and be so counterproductive and risky^16^. In this vein, it might be argued that the pain reduction we observe for iniquitous offers could be viewed as a way to hide pain related feelings and discomfort in a threatening context. However, if so, we should observe the same pain reduction in reaction to an unfavorable offer, and, even more, an interaction between Moral Valence and Personal Valence on pain ratings (with lower pain after unfavorable and iniquitous offers). Despite the attentional engagement seems the most parsimonious explanation, it has to be tested specifically with objective behavioural measures as reaction times, while our study aimed to first characterize the effects of different variables on pain. We cannot exclude that other phenomena as increased anxiety and/or arousal, induced by the unbalanced apportion, are also playing a role. However, anxiety is often reported to worsen pain-related suffering^7^, thus we could have expected a pain increase in the most unbalanced pain apportion. Indeed, a limitation of our study is that we did not tested the possible mechanisms underlying the different modulations.

It is worth noting that, in our study, a contextual cue preceded nociceptive stimuli. Consequently, expectations about receiving higher intensity or higher number of stimuli could have played a role in the modulation we observed^35–37^, leading to higher pain expectations following iniquitous offer cues. To minimize such an effect, participants were told that each stimulus was moderately intense and the inter-offer variation in the number of stimuli was very low. They were also told that the total amount of pain to be divided was very variable and, thus, the proportion presented was not informative about, or related to, the number of stimuli or their intensity.

Different factors can account for the effect of personal valence. First, we used hyper-fair offers, in which the recipient could benefit from more than 50% of the total payoff ^38^. Hyper-fair offers are considered to be unexpected and may raise social, emotional, and moral concerns^39^. Consequently, our effect may be explained by a differential attentional engagement induced by the favorable offer compared to the unfavorable ones. Our study is also in agreement with research showing that participants playing as Receivers rated pain as less intense when they believed that the Dictator had chosen to receive a higher number of shocks^18^. Knowing that the Dictator decided to endure extra pain for the benefit of the Receiver, named by the authors “kindness effect,” is likely at play in the present study and may have triggered pain reduction in the Receiver. The result is also in agreement with a study in which participants reported lower unpleasantness of painful stimuli when enduring extra pain for the benefit of their partner^40^. However, it is also possible that the analgesic effect observed is related to the favorable offer (i.e., receiving less pain than the other player) having a positive emotional valence from an egocentric perspective compared to the unfavorable offer. This could represent an intrinsic self-serving bias of the Receiver. Thus, although we cannot disambiguate whether the effect of personal valence on pain is due to attentional or emotional modulation^3,4^, we can posit that personal convenience and perceived equity can independently modulate pain perception. This result is in agreement with studies claiming that negative and positive events or inputs can differentially modulate pain perception by exacerbating and reducing pain, respectively^4,41–43^.

Regarding the fairness reports, pain and money influenced the perceived fairness of an offer differently. In the economic game, unfavorable offers were always rated as more unfair than favorable offers, whereas, in the pain game, only very iniquitous offers engendered higher perceived unfairness for unfavorable outcomes. Therefore, self-serving bias was always present in the economic game, but was only observed in the pain game when an offer was very unbalanced^44,45^. Previous work has shown that individuals become less self-centered when they have to consider the pain of others and will sacrifice more money to reduce the pain of someone else than that to reduce their own pain^46,18^. However, this hyper-altruistic effect can be diminished when central nervous system levels of dopamine are increased^47^. Moreover, other studies have demonstrated that being in pain^25^ or exposed to interoceptive stimulations like one’s own vs others’ heartbeat^26^ increases a self-centered perspective and modulates socioeconomic choices accordingly. In this study, we observed a differential contribution of self-serving bias to offer fairness evaluations depending on whether the transaction was monetary or painful. This suggests that self-serving bias is dynamic and changes with context and our beliefs. However, a limitation of our methods is that the two games were always presented in the same sequential order with the economic game presented first. The rationale is that we considered that the game involving pain perception is a distressing experience, while, as indicated by a pilot study, the economic version of the game was not. Future studies should make the economic game more engaging and then adopt a counterbalanced order of the money and pain tasks.

Concerning the lack of effect of the social valence, we observe that interacting with a partner who evaluated positively vs. negatively the participants had no clear effect on their pain and fairness ratings. A possible, although speculative, explanation is that due to the physical absence of the partner as well as the absence of a shared goal (as in^48^), our social manipulation has short-lasting and incospicuous effects. This hypothesis can be tested in future studies. To date, studies concerning the link between the social valence of an interaction and pain perception have reported mixed effects. Social rejection has been related to decreases in pain threshold, reports of pain exacerbation, reduced endogenous opioid release^8,49,50^, and the implementation of more rudimentary emotional regulation strategies, especially in patients with chronic diseases^51^. However, a hypoalgesic response contingent upon social exclusion has also been reported^12,52^.

The interpersonal manipulation used in the present study was meant to induce a sense of social rejection or acceptance by the two partners^48^. Our results showed lower mood scores for participants following negative partner feedback compared to positive feedback. Moreover, only four participants reported that they did not believe that the other partners were present. Thus, despite the lack of modulation, the interpersonal manipulation was effective in producing different attitudes toward the inclusive and exclusive Dictators.

The disambiguation of the moral and personal context of social interactions has been rarely investigated. Our results show that moral and personal aspects can differentially affect behavior and neural processing^29,53–55^. We confirm and expand upon previous results by demonstrating that personal and moral aspects have differential effects on pain perception during interpersonal interactions. We devised a novel experimental design to assess the role of social, personal, and moral variables in modulating pain and fairness perception. Our paradigm is potentially useful for testing the neural correlates of a variety of factors that modulate pain perception in healthy and clinical populations, thus paving the way to a better understanding of how contextual cues can be used for pain control.

## Methods

### Participants

Thirty-two healthy subjects (16 men and 16 women) aged 19–30 years (mean: 22.9 ± 3.3) participated in the study. To avoid recruiting psychology students, who could potentially be familiar with the subject matter, participants were selected from gyms or student unions for non-psychology or neuroscience students from the area of Rome.

All participants in this study gave their written informed consent. This study was approved by the Independent Ethics Committee Rulebook of the Scientific Institute for Research, Hospitalization, and Health Care (Protocol: CE/PROG.558, Santa Lucia Foundation, Rome, Italy), and all methods were performed in accordance with the relevant guidelines and regulations.

### Experimental design

A 2 × 2 × 4 within-subject design has been used (Table 4) to test the interactions and main effect of Social Valence x Personal Valence x Moral Valence on pain and fairness ratings.

**Table 4:** Social Valence x Personal Valence x Moral Valence 2 × 2 × 4.

		Social Acceptance		Social Rejection	
		Favorable	Unfavorable	Favorable	Unfavorable
	Very Iniquitous	8	8	8	8
	Iniquitous	8	8	8	8
	Slightly Iniquitous	8	8	8	8
	Equitable	8	8	8	8

Experimental design. A within-subject design with 8 trials for each condition has been used to test the planned comparisons. The table presents each independent variable with its respective levels and number of trials. The Social Valence is the Dictators’ attitude toward the participant and includes Social Acceptance and Social Rejection. The Personal Valence indicates the personal convenience of the offer towards the participant: Favorable offers provided more money (for the economic game) and less pain (for the Pain Dictator Game) to the participants compared to the Dictator, while Unfavorable offers, viceversa, provided less money and more pain to the participants compared to the Dictator. The Moral Valence represents the offer equity according to the payoff apportion proposed: Very Iniquitous/Iniquitous/Slightly Iniquitous/Equitable.

### Procedure

**Figure 6:**
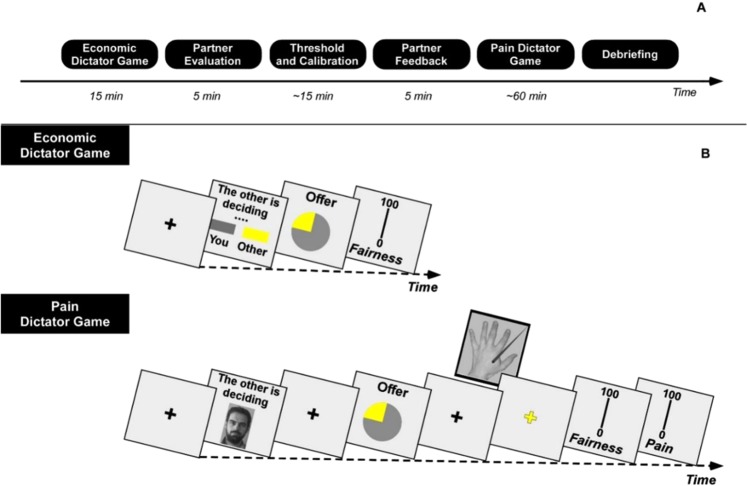
Experimental procedure. Panel A represents a timeline of the different phases. Panel B displays the details of the Economic (Panel B - top) and Pain (Panel B - bottom) Dictator Games. For the Economic Dictator Game, participants waited between 2000–4500 ms while viewing a slide stating that “the other is deciding.” Following this, participants were given an offer for 2000 ms presented as a pie chart showing the proportion of money allocated to the participant by the Dictator. For the Pain Dictator Game, participants waited between 2000–6500 ms for the Dictator’s decision. Following this, participants viewed a fixation target (i.e., black cross), which preceded a pie chart presentation of an offer lasting for 4000 ms. Following the presentation of another fixation target (i.e., black cross), a thermal nociceptive stimulus (i.e., yellow cross) was delivered with an inter-stimulus interval between 1200–1900 ms. Each presentation of the black fixation cross lasted 500 ms. Written and signed permission was obtained from each of the photographed participant.

The procedure (Fig. 6, Panel A) included: the Economic Dictator Game, Partner evaluation (see Supplementary Information), Laser Calibration, Partner’s feedback (see Supplementary Information), and Pain Dictator Game phases. Participants were informed that the aim of the study was to investigate their beliefs about other players, during a social interaction. They were also informed that they had to participate in two different games and report their feelings and judgments during each game. Before starting the game, participants were told that they had been randomly assigned to the role of Receiver in both games.

### Economic dictator game

The Dictator Game implies that one of the players, in the role of Dictator, determines how to divide a payoff between her/himself and the other player, the Receiver. The latter, instead, can only accept the Dictator’s offer and evaluate its fairness. During the phase of the Economic Dictator Game (Fig. 6, Panel B (Top)) Dictators’ identities were not disclosed to the participants. Participants were informed if they were playing with Player 1 or 2 respectively, without other information regarding the Dictators (i.e. no pictures or names). After waiting between 2000–4500 ms, an offer was presented for 2000 ms as a pie chart, which showed the proportion of the money allocated to the participant by the Dictator. Participants were not informed about the total amount of money (/pain) to be divided on each trial by the Dictator. They were told that, on the basis of the outcome of the game, a final pay-off would be added to their reimbursement. The grey and yellow areas of the pie chart represented the participants’ and Dictators’ amount respectively. Participants were asked to look at the pie chart and rate the equity of the received offer on a 0–100 scale, with 0, extremely iniquitous, and 100, extremely fair. Four different levels of moral valence were presented: very iniquitous (i.e., offers of 90/10 or 85/15), iniquitous (i.e., offers of 80/20 or 75/25), slightly iniquitous (i.e., offers of 70/30 or 65/35), and equitable (i.e., offers of 60/40 or 55/45) for each personal valence level (favorable/ unfavorable). The game consisted of 128 trials (Table 4) and was presented as two blocks of 64 trials separated by a short break. For the Economic Dictator Game, the Dictator’s attitude toward the participant (i.e., social valence) was not manipulated.

### Radiant-heat stimulation and calibration

Noxious radiant-heat stimuli were generated by an infrared neodymium yttrium aluminum perovskite (Nd:YAP) laser with a wavelength of 1.34 μm (Electronical Engineering, Florence, Italy). Laser pulses directly activate Aδ- and C-fiber nociceptive terminals located in the superficial layers of the skin^56^. The laser beam, with a diameter of 6 mm (≈ 28 mm^2^), was transmitted via an optic fiber. Each pulse, lasting 4 ms, was delivered to a 5 × 5-cm area on the dorsum of the left hand defined prior to the start of the experimental session^57^. The position of the laser beam, indicated by a He-Ne laser, was changed after each pulse in order to prevent significant changes in skin temperature or nociceptor fatigue or sensitization. Skin temperature was measured by an infrared thermometer (precision: ±0.3 °C). The intensity of the painful stimuli was chosen during the individual calibration phase by adjusting energy value (J) so that each participant rated as moderate and tolerable (rated between: 35–55). According to the method of limits, stimulus intensity was increased and decreased in steps of 0.5 J (Joules). Afterwards, a second ascending and descending series of 0.25 J steps has been administered. The series were then narrowed around the intensity that the participant rated as moderate pain sensation 50 ± 10% of times.

### Dictator game with painful stimuli

In the Pain Dictator Game (Fig. 6, Panel B (Bottom)) the offer consisted of three radiant-heat painful stimuli, delivered to the participant’s hand-dorsum. Each stimulus was delivered with a random inter-stimulus interval between 1200–1900 ms. During each trial, a 2000–6500 ms interval elapsed before participants were shown the offer. It was represented by a pie chart appearing for 4000 ms on screen. After receiving the nociceptive stimuli included in the offer, participants rated, using a 0–100 scale, the perceived pain, from no pain (0) to the worst possible pain (100), and the fairness of the offer, from extremely unfair (0) to extremely fair (100). The order of the two ratings was randomized. To maintain constant sensory input, each stimulus has always the same intensity, which was chosen during the calibration phase as the energy level (J) that the participant rated as moderate and always tolerable. The Pain Dictator Game was divided into four blocks, 32 trials each. The offer always consisted of three stimuli except for extra trials, in which the offer consisted of four. This prevent participants from noticing the fixed number of stimuli. One of these extra trials occurred in each block, and two extra trials in the second block. All of these extra trials were excluded from the statistical analyses. The behavior of the Dictator was balanced across the four blocks. The presentation of the type of offer followed a pseudo-random sequence, in which the same type of offer was never presented more than two consecutive times from the same partner.

### Data analysis

Data analysis was performed with R (R Development Core Team, 2013). We performed a linear mixed model (LMM), or “mixed-effects model”^58^, rather than the standard analysis of variance (ANOVA), in order to account for inter-individual variability in pain perception. This approach allowed us to control for different pain perception or fairness judgment baseline levels between participants, which were modeled as random intercepts. The aim is to control for differences in how the main effects and interactions were featured in each participant (i.e., random slopes of the model). Indeed, mixed-effects models provide a powerful tool for the analysis of grouped data by incorporating random effects, or additional error terms, to account for correlation between observations within the same group^58^. For pain ratings, we considered the social (rejection/acceptance), moral (very iniquitous/iniquitous/slightly/iniquitous/equitable) and personal (i.e., favorable: more money/less pain to the participant compared to the other player and unfavorable: less money/more pain to the participants compared to the other) valence of an offer as fixed effects. For perceived fairness ratings, we considered game type (i.e., Economic or Pain Dictator Game) as a predictor, along with the other predictors included in the pain model, excluding social valence, which was not manipulated in the economic game. To select the proper random effects structure, we used the method suggested by Bates *et al*.^59^. We performed a random-effects principal component analysis (rePCA function in R). We determined the dimensionality of the variance-covariance matrix of the random-effect structure, constrain correlation parameters to zero, and drop non-significant variance components and their associated correlation parameters from the model^59^. The use of this method allowed for a simple assessment of the dimensionality of the random effects distribution^59^, but it also allows to estimate model parameters in complex models with several subjects and items^59^. Models constructions followed the following steps. First, we tested a full model that included all the factors, main effects, and interactions, (Social Valence * Personal Valence * Moral Valence) as fixed effects, and as random effects as well. In accordance with rePCA results, the model was then reduced by dropping, from the random part only, by-subject effects that explained a near-zero amount of variance. Following this, we tested two Pain Dictator Game models, in which Model 1 included pain ratings and Model 2 included fairness ratings as the dependent variable. In these models, social valence was included as a regressor. Model 3, was instead aimed to compare fairness ratings between the two games, as the social valence was manipulated in the Pain Dictator Game, but not the Economic Dictator Game, the social valence was not included as a regressor for Model 3.

The resultant models:

Model 1: Pain ~ Social Valence * Personal Valence * Moral Valence + *(Social Valence* + *Personal Valence* + *Moral Valence* + *Social Valence:Personal Valence* + *Social Valence:Moral Valence* + *Personal Valence:Moral Valence|Subj)*.

Model 2: Fairness (Pain Dictator Game) ~ Social Valence * Personal Valence * Moral Valence + *(Social Valence* + *Personal Valence* + *Moral Valence* + *Social Valence:Personal Valence* + *Social Valence:Moral Valence |Subj)*.

Model 3: Fairness (Both Dictator Games) ~ Personal Valence * Moral Valence * Game + *(Personal Valence* + *Moral Valence* + *Game* + *Personal Valence:Moral Valence* + *Personal Valence:Game* + *Moral Valence:Game|Subj)*.

All the continuous variables have been centered at their means. Using the R package *lme4* (Version 1.1–13, 4), we performed an ANOVA (type III sum of square) on each model. We considered effects to be significant when p < 0.05. For significant effects, post-hoc comparisons were performed with the Tukey test.

## Supplementary information

Procedure used to provide participants with false feedback on social acceptance or rejection (interpersonal manipulation).

## Data Availability

The datasets generated during and analysed during the current study are available from the corresponding author upon request.

## References

[1] Price DD (2000). Psychological and Neural Mechanisms of the Affective Dimension of Pain. Science (80-.)..

[2] Valeriani M (2008). Seeing the pain of others while being in pain: A laser-evoked potentials study. Neuroimage.

[3] Bushnell, M. C., Čeko, M. & Low, L. A. Cognitive and emotional control of pain and its disruption in chronic pain. *Nat. Rev. Neurosci*. **14****(****7****)**, (2013). 10.1038/nrn3516 PMC4465351 23719569

[4] Roy, M. Cerebral and spinal modulation of pain by emotions and attention. In Pain, Emotion and Cognition. In (pp. 35–52) (2015).

[5] Williams AC, de C, Craig KD (2016). Updating the definition of pain. Pain.

[6] Karos K, Williams AC, de C, Meulders A, Vlaeyen JWS (2018). Pain as a threat to the social self. Pain.

[7] Rhudy JL, Meagher MW (2000). Fear and anxiety: Divergent effects on human pain thresholds. Pain.

[8] Eisenberger NI, Jarcho JM, Lieberman MD, Naliboff BD (2006). An experimental study of shared sensitivity to physical pain and social rejection. Pain.

[9] Reicherts P, Gerdes ABM, Pauli P, Wieser MJ (2013). On the mutual effects of pain and emotion: Facial pain expressions enhance pain perception and vice versa are perceived as more arousing when feeling pain TT - Über die gemeinsamen Effekte von Schmerz und Emotion: Schmerzhafte Gesichtsausdrücke verstärke. Pain.

[10] Mogil JS (2015). Social modulation of and by pain in humans and rodents. Pain.

[11] Che X, Cash R, Chung S, Fitzgerald PB, Fitzgibbon BM (2018). Investigating the influence of social support on experimental pain and related physiological arousal: A systematic review and meta-analysis. Neurosci. Biobehav. Rev..

[12] Borsook TK, MacDonald G (2010). Mildly negative social encounters reduce physical pain sensitivity. Pain.

[13] Krahé C, Springer A, Weinman JA, Fotopoulou A (2013). The Social Modulation of Pain: Others as Predictive Signals of Salience – a Systematic Review. Front. Hum. Neurosci..

[14] Mancini A, Betti V, Panasiti MS, Pavone EF, Aglioti SM (2014). Perceiving monetary loss as due to inequity reduces behavioral and cortical responses to pain. Eur. J. Neurosci..

[15] Karos K, Meulders A, Goubert L, Vlaeyen JWS (2018). The Influence of Social Threat on Pain, Aggression, and Empathy in Women. J. Pain.

[16] Karos, K. Hide your pain? The effects of social threat on. (2018).

[17] Peeters PAM, Vlaeyen JWS (2011). Feeling more pain, yet showing less: The influence of social threat on pain. J. Pain.

[18] Story GW (2015). Social redistribution of pain and money. Sci. Rep..

[19] Mattew, R. Moral Preferences, Moral Constraints, and Self-serving Biases. (1995).

[20] Fehr E, Schmidt KM (1999). A Theory of Fairness, Competition, and Cooperation*. Quartely J. Econ..

[21] Thompson L, Loewenstein G (1992). Egocentric interpretations of fairness and negotiation. Organ. Behav. Hum. Decis. Process..

[22] Roese NJ, Olson JM (2007). Better, stronger, faster: Self-serving judgment, affect regulation, and the optimal vigilance hypothesis. Perspect. Psychol. Sci..

[23] López-Martínez AE, Esteve-Zarazaga R, Ramírez-Maestre C (2008). Perceived Social Support and Coping Responses Are Independent Variables Explaining Pain Adjustment Among Chronic Pain Patients. J. Pain.

[24] Camerer, C. & Thaler, R. H. Ultimatums, Dictators and Manners. **9**, 209–219 (1995).

[25] Mancini, A., Betti, V., Panasiti, M. S., Pavone, E. F. & Aglioti, S. M. Suffering makes you egoist: Acute pain increases acceptance rates and reduces fairness during a bilateral ultimatum game. *PLoS One* **6**, (2011). 10.1371/journal.pone.0026008 PMC3192138 22022492

[26] Lenggenhager B, Azevedo RT, Mancini A, Aglioti SM (2013). Listening to your heart and feeling yourself: Effects of exposure to interoceptive signals during the ultimatum game. Exp. Brain Res..

[27] Brosnan SF, De Waal FBM (2003). Monkeys reject unequal pay. Nature.

[28] Blake PR, McAuliffe K (2011). ‘I had so much it didn’t seem fair’ Eight-year-olds reject two forms of inequity. Cognition.

[29] Corradi-Dell’Acqua C, Civai C, Rumiati RI, Fink GR (2013). Disentangling self- and fairness-related neural mechanisms involved in the ultimatum game: An fMRI study. Soc. Cogn. Affect. Neurosci..

[30] Apps MAJ, McKay R, Azevedo RT, Whitehouse H, Tsakiris M (2018). Not on my team: Medial prefrontal cortex responses to ingroup fusion and unfair monetary divisions. Brain Behav..

[31] Puglisi-Allegra S, Oliverio A (1983). Social isolation: Effects on pain threshold and stress-induced analgesia. Pharmacol. Biochem. Behav..

[32] Kavaliers M, Colwell DD, Perrot-Sinal TS (1997). Opioid and non-opioid NMDA-mediated predator-induced analgesia in mice and the effects of parasitic infection. Brain Res..

[33] Rodgers RJ, Hendrie CA (1983). Social conflict activates status-dependent endogenous analgesic or hyperalgesic mechanisms in male mice: Effects of naloxone on nociception and behaviour. Physiol. Behav..

[34] Vervoort Tine, Karos Kai, Trost Zina, Prkachin Kenneth M. (2018). Social and Interpersonal Dynamics in Pain.

[35] Sawamoto N (2000). Expectation of pain enhances responses to nonpainful somatosensory stimulation in the anterior cingulate cortex and parietal operculum/posterior insula: an event-related functional magnetic resonance imaging study. J. Neurosci..

[36] Valentini E, Martini M, Lee M, Aglioti SM, Iannetti G (2014). Seeing facial expressions enhances placebo analgesia. Pain.

[37] Fazeli S, Büchel C (2018). Pain-Related Expectation and Prediction Error Signals in the Anterior Insula Are Not Related to Aversiveness. J. Neurosci..

[38] Henrich BJ (2001). American Economic Association In Search of Homo Economicus: Behavioral Experiments in 15 Small-Scale Societies Author (s): Joseph Henrich, Robert Boyd, Samuel Bowles, Colin Camerer, Ernst Fehr, Herbert Gintis and Richard McElreath Source: The Ame. Am. Econ. Rev..

[39] Hennig-Schmidt, H., Li, Z.-yu. & Yang, C. *Why People Reject Advantageous Offers – Non- monotone Strategies in Ultimatum Bargaining*. (2004).

[40] López-Solà, M., Koban, L. & Wager, T. D. Transforming pain with prosocial meaning. *Psychosomatic Medicine*, 10.1097/psy.0000000000000609 (2018). 10.1097/PSY.0000000000000609 PMC6218300 29846310

[41] Rhudy JL, Williams AE, McCabe KM, Russell JL, Maynard LJ (2008). Emotional control of nociceptive reactions (ECON): Do affective valence and arousal play a role?. Pain.

[42] Vachon-Presseau E (2013). The stress model of chronic pain: Evidence from basal cortisol and hippocampal structure and function in humans. Brain.

[43] Valentini E, Betti V, Hu L, Aglioti SM (2013). Hypnotic modulation of pain perception and of brain activity triggered by nociceptive laser stimuli. Cortex.

[44] Babcock L, Loewenstein G (2011). Explaining Bargaining Impasse: The Role of Self-Serving Biases. J. Econ. Perspect..

[45] Trautmann ST (2009). A tractable model of process fairness under risk. J. Econ. Psychol..

[46] Crockett MJ (2015). Harm to others outweighs harm to self in moral decision making. Proc. Natl. Acad. Sci..

[47] Crockett MJ (2015). Dissociable Effects of Serotonin and Dopamine on the Valuation of Harm in Moral Decision Making. Curr. Biol..

[48] Sacheli, L. M., Candidi, M., Pavone, E. F., Tidoni, E. & Aglioti, S. M. And Yet They Act Together: Interpersonal Perception Modulates Visuo-Motor Interference and Mutual Adjustments during a Joint-Grasping Task. *PLoS One* **7**, (2012). 10.1371/journal.pone.0050223 PMC3509140 23209680

[49] Bungert M (2015). Pain processing after social exclusion and its relation to rejection sensitivity in borderline personality disorder. PLoS One.

[50] Hsu DT (2015). It still hurts: altered opioid activity in the brain during social rejection and acceptance in major depressive disorder. Mol. Psychiatry.

[51] Ponsi G, Panasiti MS, Scandola M, Aglioti SM (2016). Influence of warmth and competence on the promotion of safe in-group selection: Stereotype content model and social categorization of faces. Q. J. Exp. Psychol..

[52] Dewall, C. N. Alone but Feeling No Pain: Effects of Social Exclusion on Physical Pain Tolerance and Interpersonal Empathy. (2006). 10.1037/0022-3514.91.1.1 16834476

[53] Civai C, Crescentini C, Rustichini A, Rumiati RI (2012). Equality versus self-interest in the brain: Differential roles of anterior insula and medial prefrontal cortex. Neuroimage.

[54] Feng C (2013). The Flexible Fairness: Equality, Earned Entitlement, and Self-Interest. PLoS One.

[55] Sauermann J, Kaiser A (2010). Taking others into account: Self-interest and fairness in majority decision making. Am. J. Pol. Sci..

[56] Iannetti GD, Zambreanu L, Tracey I (2006). Similar nociceptive afferents mediate psychophysical and electrophysiological responses to heat stimulation of glabrous and hairy skin in humans. J. Physiol..

[57] Valentini E, Nicolardi V, Aglioti SM (2017). Visual reminders of death enhance nociceptive – related cortical responses and event-related alpha desynchronisation. Biol. Psychol..

[58] Garson, G. D. Fundamentals of Hierarchical Linear and Multilevel Modeling. In 3–27 (2012).

[59] Bates, D., Kliegl, R., Vasishth, S. & Baayen, H. Parsimonious Mixed Models. 1–27 (2015).

