# Author Correction: Pain perception during social interactions is modulated by self-related and moral contextual cues

**DOI:** 10.1038/s41598-020-60647-6

**Published:** 2020-02-21

**Authors:** Valentina Nicolardi, Maria Serena Panasiti, Mariagrazia D’Ippolito, Gian Luigi Pecimo, Salvatore Maria Aglioti

**Affiliations:** 1 Department of Psychology, Sapienza University of Rome, Rome, Italy; 2 Fondazione Santa Lucia, IRCCS, Rome, Italy; 3 Istituto Italiano di Tecnologia, Genova, Italy

Correction to: *Scientific Reports* 10.1038/s41598-019-56840-x, published online 08 January 2020

This Article contains an error in Reference 51, which was incorrectly given as:

Ponsi, G., Panasiti, M. S., Scandola, M. & Aglioti, S. M. Influence of warmth and competence on the promotion of safe in-group selection: Stereotype content model and social categorization of faces. *Q. J. Exp. Psychol*. **69**, 1464–1479 (2016).

The correct reference is listed below:

Ponsi, G., Monachesi, B., Panasiti, V., Aglioti, S. M., & Panasiti, M. S. Physiological and behavioral reactivity to social exclusion: a functional infrared thermal imaging study in patients with psoriasis. *Journal of neurophysiology*, **121**(1), 38–49 (2019).

