# Supplementary material for: Pain perception during social interactions is modulated by self-related and moral contextual cues
Source: Sci Rep. 2020 Jan 8;10:41. doi: 10.1038/s41598-019-56840-x (PMC6949219; doi:10.1038/s41598-019-56840-x)
Supplement: Supplementary file 1 — Procedure used to provide participants with false feedback on social acceptance or rejection (interpersonal manipulation). [file 41598_2019_56840_MOESM1_ESM.docx]

Pain perception during social interactions is modulated by self-related and moral contextual cues

Valentina Nicolardi^1,2 *^; Maria Serena Panasiti^1,2^; Mariagrazia D’Ippolito^1,2^; Gian Luigi Pecimo^1,2^; Salvatore Maria Aglioti^1,2^

^1^Department of Psychology, Sapienza University of Rome, Rome, Italy

^2^Fondazione Santa Lucia, IRCCS, Rome, Italy

*Corresponding author: [valentina.nicolardi@uniroma1.it](mailto:valentina.nicolardi@uniroma1.it)

Disclosure of conflicts of interest: The authors declare no conflicts of interest.

Category: Original article

Fundings: financial support was provided by a PRIN (Progetti di Ricerca di Rilevante Interesse Nazionale, Edit. 2015, Prot. 20159CZFJK) Grant and European Research Council (ERC) Advanced Grant 2017 (*Embodied honesty in real world and digital interactions* [*eHONESTY*], 789058) to SMA and an “Avvio alla Ricerca” Grant 2016 awarded by the Sapienza University of Rome to VN.

## *Procedure used to provide participants with false feedback on social acceptance or rejection (interpersonal manipulation)*

We implemented an interpersonal manipulation based on a previous study ^69^, which we have also found to be effective in motor interaction tasks ^58^. We induced a sense of social acceptance or rejection by providing participants with positive or negative feedback from two Dictators (Fig. 2). This process allowed us to manipulate two levels of social valence (i.e., acceptance or rejection). In order to convince the participants that our confederates were actual players whom they would meet at the end of the experimental session, we designed a special recruitment procedure in which they received an e-mail with multiple recipients (contained within a hidden Cc), including our confederates. This e-mail served as a reminder of their appointment date and time, and requested each participant to send a picture for use during games and complete a questionnaire about themselves. The questionnaire included five open questions regarding their background (i.e., family and education), future plans for the next three years, personal interests and hobbies, personality (i.e., three gifts and flaws), and three objects “you would bring on a desert island” (as in ^58^). During the experimental session and following the Economic Dictator Game, we showed the participants pictures of their two game partners and asked them to judge each partner on 10 dimensions. On a scale from 0 to 100, participants rated how kind, casual, nice, unimpressive, charming, interesting, pleasant, original, and similar to the participant their partners were, as well as how much the participant liked him or her (Fig. 2, “Partner Evaluation”). To avoid social desirability effects, we explicitly told participants that their partners were not allowed to read their evaluations and that only the Receiver (i.e., the participant) was allowed to read evaluations. Following the calibration phase (Fig. 2, “Partner Feedback”), we showed participants how the other partners had rated them using the same questionnaire. Following this, participants were asked to rate how much the received feedback elicited pleasure, nuisance, and satisfaction using a 0–100 scale. The latter ratings were used to calculate a Mood index, indicating how much each participants’ mood has been positively or negatively affected by the social manipulation. At the end of the experiment, participants filled out a manipulation check questionnaire aimed at checking the efficacy of the whole manipulation. Specifically, the following questions were asked: 1-“Have you noticed any difference in the behavior of your partners?”; 2-“If yes, what do you think is the reason?;” 3-“Have you noticed or felt something strange during the procedure?;” 4-“Did you feel deceived by the experimenters?;” 5-“Have you ever had the sensation that your partner was not participating in the game?;” and 6-“Any other comments?”. Any participant who answered affirmatively to question 4 and 5, or who reported doubts regarding the procedure’s reliability at question 3, were considered as “non-believers”.

## *Manipulation check*

To test the efficacy of manipulation, we analyzed participants’ responses to the manipulation check questionnaire and mood reports completed after receiving partner feedback. In the manipulation check questionnaire, only four of thirty-two participants reported being deeply doubtful during the procedure and suspicious about whether their two partners were present. These participants were classified as non-believers. Considering the lack of balance between the number of believing and non-believing participants, we decided to test our models with and without the non-believers to determine if the presence of non-believers affected the results. A t-test was used to determine whether there were significant differences between mood reports following social rejection or acceptance by the Dictators. We calculated an index of general mood by subtracting the nuisance score from the sum of the pleasure and satisfaction scores. We then analyzed the general mood index.
